# Inhibition of BET Proteins and Histone Deacetylase (HDACs): Crossing Roads in Cancer Therapy

**DOI:** 10.3390/cancers11030304

**Published:** 2019-03-05

**Authors:** Gloria Manzotti, Alessia Ciarrocchi, Valentina Sancisi

**Affiliations:** Laboratory of Translational Research, Azienda Unità Sanitaria Locale-IRCCS di Reggio Emilia, 42122 Reggio Emilia, Italy; gloria.manzotti@ausl.re.it (G.M.); alessia.ciarrocchi@ausl.re.it (A.C.)

**Keywords:** epigenetic drugs, histone modifications, BET protein, HDAC, combination therapy, cancer

## Abstract

Histone DeACetylases (HDACs) are enzymes that remove acetyl groups from histones and other proteins, regulating the expression of target genes. Pharmacological inhibition of these enzymes re-shapes chromatin acetylation status, confusing boundaries between transcriptionally active and quiescent chromatin. This results in reinducing expression of silent genes while repressing highly transcribed genes. Bromodomain and Extraterminal domain (BET) proteins are readers of acetylated chromatin status and accumulate on transcriptionally active regulatory elements where they serve as scaffold for the building of transcription-promoting complexes. The expression of many well-known oncogenes relies on BET proteins function, indicating BET inhibition as a strategy to counteract their activity. BETi and HDACi share many common targets and affect similar cellular processes to the point that combined inhibition of both these classes of proteins is regarded as a strategy to improve the effectiveness of these drugs in cancer. In this work, we aim to discuss the molecular basis of the interplay between HDAC and BET proteins, pointing at chromatin acetylation as a crucial node of their functional interaction. We will also describe the state of the art of their dual inhibition in cancer therapy. Finally, starting from their mechanism of action we will provide a speculative perspective on how these drugs may be employed in combination with standard therapies to improve effectiveness and/or overcome resistance.

## 1. Introduction

Histone acetylation changes chromatin organization and largely affects gene expression regulation. The chromatin acetylation status is regulated by three main protein families. The “Writers” are Histone Acetyl Transferase (HATs). They add acetyl groups to the lysine residues on target proteins including histones, leading to loosening of chromatin conformation and promoting transcription. The “Erasers” are Histone DeACetylase (HDACs). They remove acetyl groups from histone and non-histone proteins returning chromatin to a less accessible conformation and restraining transcription. Besides, high-resolution mass spectrometry studies identified 3600 lysine acetylation sites on over 1700 non-histone targets, which are preferential components of large macromolecular complexes, such as chromatin remodeling, cell cycle, and splicing [1]. The “Readers” are proteins that bind acetylated histones. The Bromodomain and Extraterminal domain (BET) family is the best characterized class of acetylation Readers. By accumulating on hyper-acetylated chromatin regions as active promoters or enhancers, these proteins serve as scaffolds for the recruitment of transcription factors and multi-protein complexes that promote transcription of target genes [2].

Cancer development and progression heavily rely on gene expression reprogramming [3,4]. Since they have a crucial role in transcription regulation, both HDAC and BET proteins have been considered as potential targets for anticancer strategies. Several HDAC and BET protein inhibitors (HDACi, BETi) have been developed and tested into preclinical and clinical models, entering into clinical application in many cancer settings. Here we aim to summarize the employment of these drugs in the cure of cancer, discussing also their potential interplay with other drugs.

## 2. HDAC Proteins

The HDAC superfamily comprises eleven components divided in classes (I, IIa, IIb, and IV) and seven sirtuins (class III) [5,6,7]. Historically, HDACs activity has been linked to gene repression. These enzymes accumulate on repressed genes [8], reverting HATs effects, leading to histone deacetylation, chromatin condensation, and gene repression. They are also part of complexes involved in transcription silencing, such as the HDAC/mSin3/N-CoR/SMRT repressor complex [9]. However, several studies showed that inhibition of HDACs causes the direct repression of many genes [10,11,12] and genome-wide studies [13] demonstrated that HDACs are preferentially bound to active genes. In particular, HDAC1 and HDAC3 are mainly recruited on promoters while HDAC2 and HDAC6 are present both on promoters and gene bodies. These observations highlight the fact that HDACs do not always repress transcription. For example, HDAC3 functions as a co-repressor when targeted on promoters [14], but it is necessary for the transcriptional activation of at least one class of retinoic acid response elements [14]. Also, gene expression profiling studies, conducted on colon cancer cell lines, showed that the number of up and down-regulated genes, after HDACi-treatment, is comparable [15]. This dual behavior can have multiple and non-mutually exclusive explanations. On one hand, HDACs may down-regulate transcription of transcriptional repressors, leading to the indirect induction of gene expression [16]. On the other hand, HDACs may post-translationally modify the acetylation status of non-histone transcriptional regulators, affecting their activity [17,18]. More generally, as HDACs are required to maintain the homeostasis of the histone acetylation landscape throughout the genome, they are highly present also along gene bodies and intergenic regions, cooperating to functionally differentiate these regions from enhancers and promoters [13]. If HDACs function is impaired, by the use of HDACi, the acetylation enrichment that usually characterizes active promoters and enhancers no longer differentiate these regions from the rest of the genome. As a consequence of the global increase of acetylation, there is a redistribution of histone readers leading to changes in the binding of also other proteins. In particular, Greer et al [19] demonstrated that HDACi block the transition of RNA PolII into productive elongation and this pausing is dependent on HSP90 and NELF activity.

As previously mentioned, the overall effect of HDACs on gene expression regulation requires both histone and non-histone acetylation [20]. A large number of transcription factors (TFs) and chromatin regulating proteins have been shown to be acetylated and controlled by HDACs. Acetylation can affect TFs function at multiple levels, by changing their protein–protein interactions, by affecting protein turnover, localization, and DNA-binding ability [21]. Also, depending on the protein and acetylation site, acetyl group may exert different, often opposite, effects on TF transcriptional activity [22,23,24].

Besides, HDACs affect many non-transcription related pathways. Analysis of acetylome highlighted a relevant role for acetylation homeostasis maintaining in many crucial processes, including DNA repair, nuclear transport cytoskeleton organization, ribosome, and chaperone activity [1,25].

Furthermore, recent evidence identifies a crucial role for acetylation and HDAC in metabolism and mitochondrial function [26].

HDACs have been linked to oncogenesis through multiple mechanisms, to date it is known that there is more than one mechanism through which HDACs play their role in carcinogenesis. Many studies demonstrated that both through over-expression [27,28,29,30,31,32] or aberrant interaction with transcriptional regulators [33,34,35,36] HDACs mediate the transcriptional repression of oncosuppressor genes [27,28,29,30,31,33,34,35,36]. Furthermore, different HDACs have shown to have cell context specific roles and to play different pro or anti-cancer activity depending on tumor type. The differential role of HDACs and the implication of HDAC targeting depending on the context have been extensively reviewed by Wawruszak et al. [36].

**Figure 1:**
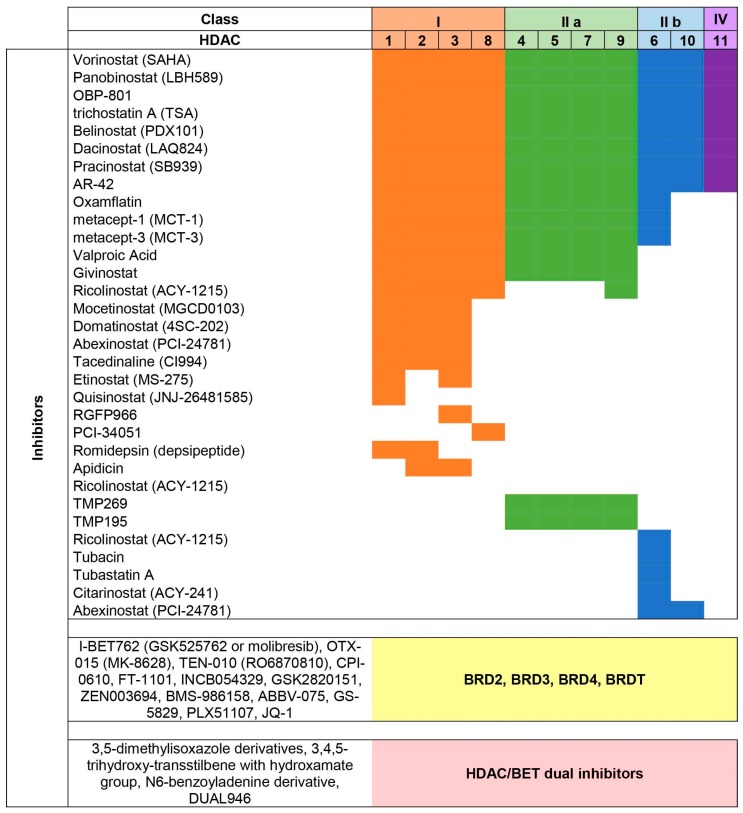
Specificity of various Histone DeACetylase (HDAC) inhibitors for members of the four classes of HDACs. Compounds inhibiting Bromodomain and Extraterminal domain (BET) proteins and dual inhibitors are also showed.

HDAC inhibitors (HDACi) can be classified according to their chemical structure into four groups: pan-inhibitors hydroxamic acids (i.e., TSA, SAHA, panobinostat), class I and IIa inhibitors aliphatic acids (i.e., VPA, butyric acids), class I and IV inhibitors benzamides (i.e., mocetinostat), and class I and IIb inhibitors cyclic tetrapeptides (i.e., romidepsin) (Figure 1) [37,38,39].

Several HDACi demonstrated clinical effectiveness in different types of hematological malignancies [40,41,42]. On the contrary, benefits of monotherapy in solid tumors are uncertain [43,44,45,46] even if there are some progresses in preclinical models.

## 3. BET Proteins

The BET family comprises four members (BRD2, BRD3, BRD4, and BRDT), which share two N-terminal tandem bromodomains and a C-terminal Extraterminal motif. These proteins bind acetylated histones [47] and recruit other proteins to form complexes that stimulate transcription initiation and elongation [2,48].

BRD4 is the most characterized member of this family and heavily implicated in transcriptional regulation and tumorigenesis [49]. BRD4 localizes on both gene promoters and enhancers and has been shown to accumulate specifically on regulatory regions termed “super-enhancers” [50].

Many oncogenes regulating cancer cell proliferation, resistance to apoptosis, and aggressiveness are under the control of BRD4. c-MYC is the first oncogene that has been described to be regulated by BRD4, both in solid tumors and hematological malignancies [51,52], providing a rationale for the development of pharmacological inhibitors of BET proteins. Besides c-MYC, several other oncogenes have been described to be under the control of BRD4, including FOSL1 (FRA-1), BCL-2, RUNX2, and c-KIT [53,54,55,56].

BRD4 is an important player in multiple steps of the transcription hierarchy: scaffolding platform for several TF (variable based on the cellular model), recruiter of the transcriptional machinery, active modifier through its phosphorylation and acetylation activity. P-TEFb is CDK/Cyc complex consisting of CDK9 and Cyclin T1 or T2 or K, its function is essentially to induce the release of the promoter-proximal paused RNA PolII thus triggering the synthesis of full-length mRNA [57,58,59]. The majority of P-TEFb is sequestrated in the inactive complex 7SK formed by 7SKsnRNA, HEXIM1, MePCE and LARP7 [60]. BRD4 interacts with P-TEFb, releasing it from 7SK inhibitory complex, and recruits P-TEFb on gene promoters, where CDK9 phosphorylates RNA-Pol II and promotes elongation of mRNAs [61].

In addition, BRD4 has been shown to regulate molecular mechanisms related to the repair of damaged DNA and to be implicated in aberrant telomere regulation in cancer, highlighting the diversity of functions of this protein in carcinogenesis [62,63].

Supporting the role of BET proteins in tumorigenesis, a translocation between NUTM1 gene and BRD3 or BRD4 has been reported in 75% of cases of NUT-midline carcinoma, leading to a formation of a fusion protein that is thought to promote transcription of oncogenes [64].

The first BET inhibitors (BETi) to be synthetized have been the benzodiazepine I-BET762 (also known as GSK525762) and the thienodiazepine JQ1, followed by others, including I-BET 151 (GSK1210151A), OTX-015, TEN-010, ZEN003694, GS-5829, and CPI-0610 (Figure 1) [2,65,66].

In 2015, the results of phase I clinical trial for the treatment with OTX-015 of patients affected by acute leukemia, lymphoma or multiple myeloma (MM) have been published [67,68], reporting tolerable toxicity and encouraging efficacy. Other trials evaluating different BETi in different cancer settings are currently undergoing, with still uncertain results.

## 4. Molecular Interplay Between HDAC and BET Proteins and Dual Targeting Inhibition Strategies

HDACs control the levels of acetylation of lysines residues on histone tails, thus shaping the acetylation profile of the genome [1], BET proteins read the histone acetylation code facilitating the activation of specific target genes [2]. Thus, it is not surprising that inhibition of these two different classes of proteins may converge on the same molecular pathways and targets. Direct and indirect interplay between HDAC and BET proteins have been described.

First of all, HDAC inhibition induces profound changes in the chromatin acetylation profile increasing the overall acetylation status also in generally deprived or low-density acetylated regions like intergenic and gene body regions [10,11,12,13]. The redistribution of acetylation throughout the genome affects recruitment of BET proteins, delocalizing them from regulatory elements to new acetylated sites and restraining their positive effect on gene transcription.

**Figure 2:**
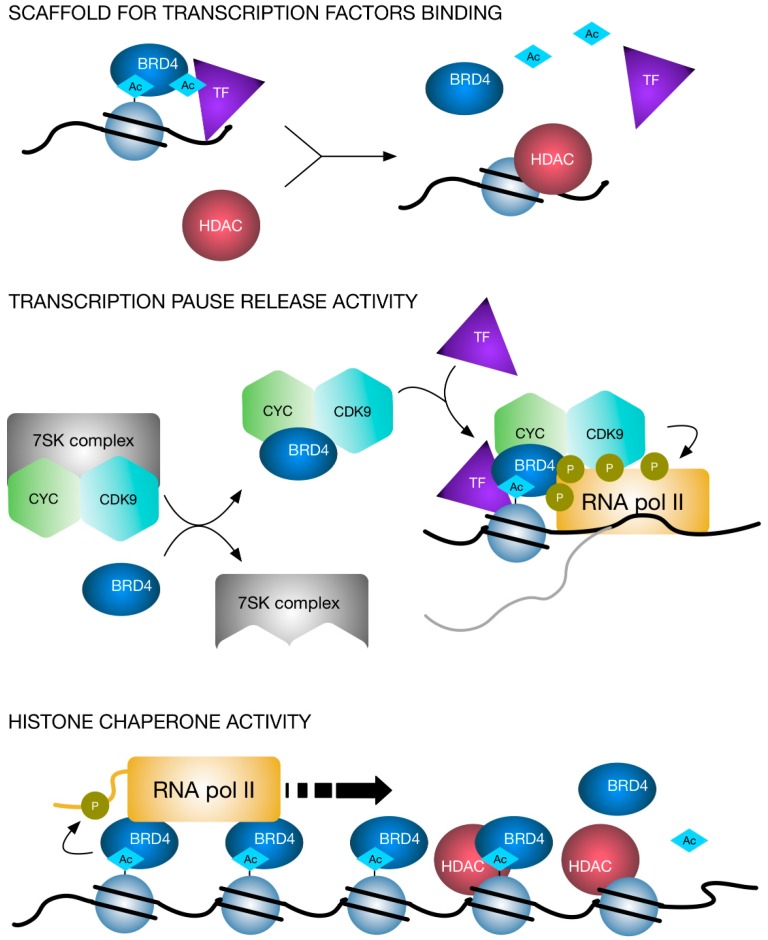
Model of molecular interplay between HDAC and BET proteins. BRD4 is a reader of acetylated histones and can bind also to other acetylated proteins such as transcription factor (TF) or co-factors, thus functioning as a scaffold unit. Moreover, BRD4 interacts with P-TEFb, releasing it from 7SK inhibitory complex and recruiting P-TEFb to promote transcriptional pause release. BRD4 has also histone-chaperone activity: it assists the passage of RNA PolII through acetylated nucleosomes. HDACs can impair BRD4 functions by removing acetylated residues from histones and other proteins. This leads to a change in the BRD4 presence on nucleosomes and on chromatin-associated protein complexes.

Secondarily, it is established that BETi affects preferentially transcription of oncogenes to which cancer cells are addicted. This is possible because of super-enhancers, which are central for cancer cells survival and are specifically dependent on BRD4 activity [49]. Also, HDACi preferentially affect highly expressed oncogenic amplicons, through induction of RNA PolII elongation-pause at genome-wide level. This leads to transcriptional repression, affecting especially transcripts which require efficient transitioning from initiation to elongation phase and this event is highly related to acetylation of histones and other transcription regulators [11]. Finally, BRD4 has histone-chaperone activity. Exploiting its interaction with acetylated histones, it assists the passage of RNA PolII through hyperacetylated nucleosomes [69] (Figure 2).

Additional evidence of an indirect functional interaction between HDACs and BET proteins has been recently provided. Hu et al. [70] demonstrated that, upon stress stimuli, PP1a dephosphorylates H3S10ph, thus enabling the nucleosome to be deacetylated by HDAC1/2/3. Decrease in histone acetylation leads to release of the chromatin-associated BRD4. In the meantime, PP2B cooperates with PP1a to dephosphorylate CDK9, leading to the liberation of P-TEFb from the inactive complex [70].

Another possible mechanism of interplay between HDAC and BRD4 is at the level of the N-CoR/HDAC3 complex. Fu et al. [71] demonstrated that HEXIM1 binds to N-CoR enabling HDAC3 to deacetylate CDK9. This modification restrains CDK9-dependent phosphorylation of the RNA Pol II CTD domain, counteracting the BRD4-mediated activation effect on RNA-PolII pausing release at the level of TSS.

Further indirect evidence of a functional interplay between HDACs and BET proteins are provided by Pinz et al. [72] and are linked to STAT proteins. These authors demonstrated that HDACi block STAT5 transcriptional activity by interfering with the function of BRD2, likely preventing it from recruiting and stabilizing the transcriptional machinery. Their data indicate that HDACi-mediated inhibition of STAT5 target genes expression is independent of the STAT5 acetylated status and is not even associated to disruption of STAT5-containing protein complexes. Indeed, the effect is due to the delocalization of BRD2 from the soluble to the insoluble chromatin fraction thus limiting its availability to support STAT5-mediated transcription [72]. 

Several studies have reported the efficacy of dual inhibition of HDAC and BET proteins to induce growth arrest and apoptosis in different types of tumor cell models [73,74,75,76,77]. In murine c-Myc-induced lymphoma, Bhadury and collegues [73], found that BETi effects are mediated by transcriptional changes genetically and functionally linked to that of HDACi. Moreover, comparing their gene expression signatures (GES) with those publicly available, they noticed that about 25 to 30% of genes induced by HDACi are also induced by BETi, this was not observed among genes suppressed. In neuroblastoma cell lines [74] and acute myeloid leukemia (AML) cells (both cell lines and patients-derived blood peripheral cells) [75], co-treatment with panobinostat and JQ1 is able to induce, respectively, apoptosis and growth inhibition more effectively than the two treatments alone. Moreover, treatment had no significant effects on normal cells and blocks tumor progression, improving survival of mice engrafted with cancer cells. Again JQ1, in combination with SAHA (a pan-HDACi) causes a potent and sustained antitumoral response, in both in vitro and in vivo models of pancreatic ductal adenocarcinoma (PDAC). In particular, Mazur et al [76] identified the activation of p57 as a key mediator of cell death, both via transcriptional induction and decreased phosphorylation (via AKT). In urothelial carcinoma cell line, JQ1 combined with romidepsin demonstrated a synergistic effect on apoptosis induction [77].

A combination of panobinostat and I-BET151 causes a strong induction of apoptosis and cell cycle arrest of melanoma cell lines and cells from melanoma patients resistant to BRAFi. In particular, this effect is achieved through the mitochondrial, caspase-dependent pathway and involves the down-regulation of Hippo/Yap and AKT pathways [78]. Similarly, panobinostat and OTX-015 reduce proliferation and enhance caspase-mediated apoptosis in glioblastoma cells, in particular phosphorylation and glycolysis are suppressed leading cells to an energy crisis and a profound stress response [79].

Different combination of HDACi and BETi have been reported to target STAT5 transcriptional activity at a step subsequent to its binding to DNA, but the molecular mechanism remains unclear [72,80,81,82].

These and other works provide evidence to support the idea of combined therapy, but some researchers went further and tried to produce a single molecule able to inhibit both HDAC and BET protein [83,84,85,86]. Basically, the idea is to combine the active group of BET and HDAC inhibitors into one molecule in order to develop new and more efficient drugs.

Zhang et al. [83] have synthetized a series of novel 3,5-dimethylisoxazole derivatives as BRD4/HDAC dual inhibitors, demonstrating their antiproliferative effects on chronic myeloid leukemia (CML) and AML cell lines. Shao et al. [84] reported a 3,4,5-trihydroxy-transstilbene with a hydroxamate group to be more effective than combination of BETi and HDACi in killing c-Myc-induced murine cell lymphoma.

Atkinson et al. [85] reported a dual active BRD/HDAC inhibitor (DUAL946): a BET active tetrahydroquinoline (THQ) core with a hydroxamic acid HDAC inhibitor motif. They showed inhibitory activity both in in vitro biochemical assays and in cell cultures. Amemiya et al. [86] synthetized a N6-benzoyladenine derivative able to inhibit BRD4 and HDAC activity in vitro. They demonstrated its ability to inhibit growth and induce differentiation of AML cell lines.

These dual BET/HDAC inhibitors were shown to be efficacious in vitro, but further investigations are needed to define effectiveness and specificity of dual inhibitors. Currently, none of these drugs has been employed on human patients (Figure 1) [83,84,85,86].

## 5. Combining HDAC Inhibitors and BET Protein Inhibitors with Other Anti-Cancer Drugs

As previously discussed, HDACi and BETi demonstrated clinical efficacy in hematological malignancies, while efficacy in solid tumors as monotherapy seems to be limited. The strategy to combine together these two classes of epigenetic drugs holds great potential, as shown in different cancer models and already discussed in Section 4. Unfortunately, to date no clinical trials have been started to investigate the possibility to combine these two classes of anti-cancer drugs in patients. However, the ability of HDAC and BET proteins to interfere with DNA repair mechanisms and to deeply impact on chromatin organization and gene expression, modulating the levels of crucial factors for cell cycle progression, apoptosis and cell differentiation, provided a rationale to combine these drugs with other currently available anticancer therapies. Here we provide a schematic summary of the most promising combination strategies employing either HDACi of BETi in the oncological setting (see also Table 1; Table 2).

### 5.1. Combination of HDACi with DNA Damaging Agents and PARP Inhibitors

Induction of DNA damage is a common mechanism of action of radiotherapy and of several classes of conventional chemotherapeutic agents, including platinum-based compounds, topoisomerase inhibitors, alkylating agents and pyrimidine analogues. DNA damage leads to activation of DNA repair pathways through non-homologous-end joining (NHEJ) and/or homologous recombination (HR). Failure to repair DNA damage induces cell death through mitochondrial apoptosis activation [87]. Furthermore, inhibitors of the DNA repair protein poly ADP ribose polymerase (PARPi) have proven efficacy in the treatment of cancer types already deficient for DNA repair (e.g., BRCA-mutated ovarian cancer) [88].

HDACi have been shown to synergize with PARPi and DNA-damaging agents through multiple mechanisms, including the induction of DNA damage [89], attenuation of HR protein ATM activity [90], inhibition of both NHEJ and HR systems [91], induction of p53 acetylation and activity [92], downregulation of anti-apoptotic proteins, and upregulation of pro-apoptotic proteins [93,94].

A number of papers reported pre-clinical evidence of synergism between DNA-damaging agents and HDACi in both hematological and solid cancer models [95,96,97,98]. Additionally, several papers reported that the ability of HDACi to impair the DNA repair systems has the effect to sensitize cancer cells to PARPi treatment [99,100]. HDACi can also sensitize cancer cells to radiation treatment in different solid tumors, including glioblastoma multiforme, melanoma, head and neck squamous cell carcinoma (HNSCC), prostate carcinoma, colorectal carcinoma, and non-small cell lung cancer (NSCLC) [101,102,103,104].

Phase I and phase II clinical trials demonstrated enhanced efficacy of vorinostat in combination with carboplatin-paclitaxel compared to placebo-carboplatin-paclitaxel in solid tumors but also increased toxicity [105,106]. A modest clinical benefit was also showed by combining topoisomerase inhibitors doxorubicin with vorinostat or epirubicin with valproic acid [107,108]. Notably, in the case of doxorubicin-vorinostat combination, HDAC2 expression was identified as a predictive biomarker of response [107,108]. There are several ongoing and completed clinical trials combining HDACi with radiotherapy or with radiotherapy and chemotherapy. The results of Phase I trials have been published for the combination of vorinostat with short-term palliative radiotherapy for gastrointestinal carcinoma [109], for the combination of panobinostat with radiotherapy in high grade glioma [110], for the combination of valproic acid with radiotherapy and capecitabine in colorectal cancer and pancreatic cancer [111,112]. In all these studies the combination of chemo/radiotherapy with HDACi has been well tolerated and showed encouraging efficacy.

### 5.2. Combination of HDACi with RTK Pathway Inhibitors

Receptor tyrosine kinases (RTK) are a family of cell surface receptors that activate intracellular signaling pathways in response to extracellular ligands. In cancer, aberrant and/or constitutive activation of these receptors results in uncontrolled proliferation and resistance to apoptosis. Small molecule inhibitors and monoclonal antibodies against various member of this family have proven efficacy in different cancer settings, such as EGFR inhibitors in KRAS-wild type lung cancer and HER2 antibodies in HER2 positive breast cancer [125].

Starting from membrane RTKs, two main signaling cascades transduce to the nucleus pro-proliferative and pro-survival signals: the RAS/RAF/MAPK/ERK pathway and the PI3K/AKT/mTOR pathway. These pathways are frequently aberrantly activated in cancer due to activating mutations in RTK or in downstream members, like the small GTPase RAS proteins, the RAF kinases or due to inactivating mutations in oncosuppressive regulators such as PTEN. Growth signals are transmitted to the nucleus where the transcription of pro-oncogenic proteins is activated, including c-MYC and cyclin D1. Specific inhibitors of several members of these pathways have been developed and implemented as anti-cancer compounds. However, the insurgence of resistance through various mechanisms often occurs, requiring the development of more effective therapeutic strategies [126].

HDACi have been shown to have a synergistic or additive effect with multiple different inhibitors of the RTK pathway, through their ability to downregulate expression at transcriptional and/or post-translational level of RTKs, including EGFR, HER2, and HER3 [127,128]. These drugs can restore sensitivity to RTKi also by downregulating the expression of downstream effectors of RTK pathway like c-MYC and cyclin D1 [129,130] or downregulating the expression of key proteins in alternative mitogenic pathways, including MET and AKT [131]. HDACi can also sensitize cancer cells to RTKi treatment by upregulating pro-apoptotic proteins (e.g., BIM) [132] and/or downregulating anti-apoptotic proteins (e.g., survivin and XIAP) [133].

The ability of HDACi to attenuate MAPK and AKT pathways [134] and to suppress pro-survival while inducing pro-apoptotic signals [135] provides a rationale for combination of these drugs also with mTORi. Additional mechanisms have been described to underline synergism between these two classes of anti-cancer drugs, including inhibition of pro-angiogenic signals through HIF1-α protein repression [136], induction of catastrophic oxidative stress [137], and inhibition of c-MYC/E2F activity [138].

Phase I clinical studies evaluating HDACi in combination with inhibitors of RTK signaling have shown good tolerability and phase II studies are ongoing [119,121,122]. The results of phase I clinical trials for the treatment of advanced solid and hematological malignancies with a combination of vorinostat with mTORi ridaforolimus and sirolimus have been published. These studies reported good tolerability of the combination treatment and encouraging efficacy in advanced renal carcinoma, refractory Hodgkin lymphoma, perivascular epithelioid tumor, and hepatocellular carcinoma [116,117].

### 5.3. Combination of HDACi with Proteasome Inhibitors

Cancer cells show enhanced proliferation rate compared to normal cells and thus they require also a fully efficient system to warrant protein degradation and turn-over. Accordingly, it has been shown that cancer cells are particular sensitive to inhibition of protein-degradation proteasome complex [139]. Bortezomib and carfilzomib are two small molecule proteasome inhibitors approved by FDA for treatment of MM. 

The combination of HDACi with proteasome inhibitors has been investigated in many pre-clinical studies involving different tumor models. In 2004, Pei et al. [140] showed that combination of bortezomib with HDACi induced mitochondrial dysfunction and ROS production in MM cells. The combination of these two drugs showed synergistic activity both in vitro and in vivo through a ROS-dependent mechanism. Induction of apoptosis through generation of ROS and endoplasmic reticulum (ER) stress has been reported also in other cancer types treated with combinations of bortezomib and different HDACi [141,142].

Treatment of cancer cells with proteasome inhibitors stimulates the retention of ubiquitin-conjugated proteins into structures termed aggresomes that participate in mechanisms of survival in response to proteasome inhibition. Combination of bortezomib with HDACi has been shown to reduce aggresome formation, to induce ER stress and enhance apoptosis in MM and pancreatic cancer cells [143,144].

Additionally, class I HDAC inhibitor, MGCD0103 has been shown to have an antiproliferative effect in Hodgkin lymphoma cell lines and to activate Nuclear factor-kappa B (NF-κB). Proteasome inhibitors-mediated NF-κB downregulation enhances MGCD0103-induced cell death, highlighting the presence of a further mechanism of synergy [145]. 

Phase I clinical trials of HDACi with proteasome inhibitors for the treatment of MM and various solid tumors demonstrated that this combination is well tolerated [113,114,124]. However, a phase II trial of vorinostat in combination with bortezomib in glioblastoma was terminated due lack of efficacy [115].

### 5.4. Combination of HDACi with TRAIL

TNF-related apoptosis-inducing ligand (TRAIL) induces cell death in a wide range of cancer cells but not in normal cells, through binding to death receptors TRAIL-R1 (DR4) and TRAIL-R2 (DR5/TRICK2) and triggering apoptosis by the extrinsic pathway [146,147]. TRAIL-induced apoptosis required the formation of a death-inducing signaling complex (DISC [148]) and can be attenuated by inhibitory proteins, such as FLIP [149]. TRAIL and agonistic anti-TRAIL receptor (TRAIL-R) antibodies activity in inducing apoptosis in preclinical settings has candidate these agents as promising anticancer drugs.

HDACi have been shown to synergize with TRAIL in inducing apoptosis in a number of different cancer types, through downregulation of anti-apoptotic proteins such as FLIP [150], upregulation of TRAIL-R [151,152], and enhancing the formation of the DISC complex [153].

A monoclonal antibody directed against the extracellular domain of human TRAIL (tumor necrosis factor-related apoptosis-inducing ligand) receptor 2 (TR-2), that mimics TRAIL-mediated activation of the receptor and induces cell death, has been developed and is currently in a phase I clinical trial in combination with bortezomib or vorinostat for the treatment of various types of lymphoma.

### 5.5. Combination of HDACi with Hormone Therapy

Therapeutic strategies inhibiting estrogen and androgen signaling have proven efficacy in breast and prostate cancer respectively and have entered clinical practice [154].

In preclinical breast cancer models, HDACi have been shown to induce estrogen receptor-alpha (ERα) and aromatase expression in ERα-negative cells, leading to increased sensitivity to tamoxifen and aromatase inhibitor letrozole [155,156,157]. In addition, treatment with HDACi entinostat has been shown to counteract acquired resistance to letrozole through modulation of HER2 expression and activity [158].

In 2011, the results of a phase II clinical trial evaluating vorinostat in combination with tamoxifen for the treatment of hormone therapy-resistant breast cancer have been published [118]. This study showed that vorinostat-tamoxifen combination is well tolerated and exhibited encouraging activity in reversing hormone resistance. In addition, HDAC2 expression has been identified as a predictive marker of response [118].

In prostate carcinoma models, HDACi have been shown to have an opposite effect of downregulating androgen receptor (AR) expression and AR-dependent signaling. This activity has been shown to sensitize prostate cancer cells to androgen deprivation and to synergistically inhibit proliferation, in particular in ERG-positive prostate cancer [159,160].

Recently, the results of a phase I/II trial for the treatment with panobinostat in combination with anti-androgen bicalutamide in castration-resistant prostate carcinoma have been published [120]. Panobinostat induced tolerable toxicity and increased sensitivity to bicalutamide with clinical benefit [120].

### 5.6. Combination of HDACi with Immune Checkpoint Inhibitors

Immune checkpoints are receptor/ligand connections that restrain cytotoxic T lymphocytes activation against cancer cells (e.g., PD1/PDL1 and CTLA4/B7-1/B7-2). These connections can be targeted by monoclonal antibodies that, inhibiting the immune checkpoints, stimulate the cytotoxic activity of T lymphocytes against cancer cells. This strategy is becoming part of therapeutic options for several cancer types, including NSCLC and melanoma [161].

HDACi have been shown to have immunomodulatory activity, providing a rationale for the use in combination with immune checkpoint inhibitors [162]. HDACi promote tumor cell-specific apoptosis and the presence of dead cancer cells is essential for antigen presenting cells (APC)-mediated activation of cytotoxic T cells, leading to enhanced response of immune-stimulatory therapies [163]. On the contrary, HDACi can inhibit apoptosis in T cells, preventing activation-induced cell death and enhancing CD4+ T cells infiltration and anti-cancer activity [164].

Two papers reported that HDACi treatment enhanced the effect of anti-PD1 immunotherapy in melanoma through diverging molecular mechanisms: Woods and colleagues [165] found that inhibition of class I HDAC increases the expression of PD-L1 and PD-L2 ligands, while Booth and colleagues [166] observed a downregulation of PD-L1 and PD-L2 and an upregulation of class I MHC protein MHCA, following treatment with pan-HDAC inhibitors.

In addition, it has been shown that HDACi can impair myeloid-derived suppressor cells (MDSC), a population of immune cells involved in resistance to immune checkpoint blockade. Inhibiting the function of MDSC, HDACi improve the efficacy of both anti-PD1 and anti-CTLA4 anticancer therapy [167,168].

There are several ongoing clinical trials for combinations of HDACi with immunotherapic agents. Recently, the results of a phase I/II study for the combination of entinostat with interleukin-2 in metastatic renal carcinoma have been published. This study revealed the promising clinical activity of this combination [123].

### 5.7. Combination of BETi with DNA-Damaging Agents and PARPi

As already mentioned, DNA-damaging agents, including cisplatin and carboplatin, are the basis of therapeutic regimens for a number of oncologic pathologies [87].

Klingbeil at al. [169] demonstrated that JQ1 treatment sensitize NSCLC cells to pro-apoptotic agents, including cisplatin, by downregulating anti-apoptotic proteins XIAP and FLIP. They also demonstrated that JQ1 has synergistic effect with cisplatin both in vitro and in vivo.

In 2017, a screen on a large panel of small cell lung cancer (SCLC) cell lines aimed to identify drugs that can be used in combination with standard-of-care etoposide/carboplatin therapy. This study showed that BET inhibitor MK-8628 can enhance cell killing in 20–25% of the considered cell lines [170]. 

An additive or synergistic effect of JQ1 in combination with cisplatin has been observed also in malignant pleural mesothelioma cell lines, associated with downregulation of c-MYC and FRA-1 (FOSL1) [171].

In ovarian cancer, aldehyde dehydrogenase (ALDH) activity due to ALDH1A1 gene expression has been associated with stemness and drug resistance [172]. Yokoyama et al demonstrated that ALDH1A1 gene is under the control of BRD4 and that treatment with BETi reduces ALDH activity and improves efficacy of cisplatin treatment in an orthotopic mouse model of ovarian cancer [172].

A phase I/II clinical trial for the treatment of acute myeloid leukemia with a combination of OTX-015 with azacytidine is currently ongoing.

BETi have been shown also to impair the HR pathway by directly downregulating BRCA1 and RAD51 expression, thus mimicking the presence of BRCA mutations. This finding has provided a rationale to use BETi to sensitize HR proficient cancer cells to treatment with PARPi, showing pre-clinical efficacy in breast and ovarian cancer models [173,174,175].

### 5.8. Combination of BETi with RTK Pathway Inhibitors

Due to the ability of BETi to downregulate the expression of different oncogenes belonging to the pathways activates by RTK, many works investigated the possibility to combine BETi with compounds targeting RTK pathway members to increase efficacy and overcome mechanisms of resistance.

It has been shown that in HNSCC resistance to cetuximab, a monoclonal antibody against EGFR, is supported by the expression of alternative RTKs, including HER3, MET, and AXL [176]. In this setting, BETi have been shown to abrogate cell viability of cetuximab resistant cells by downregulating the expression of alternative RTKs. The combined treatment has been shown to delay the insurgence of resistance to cetuximab in the patients-derived xenografts [176]. A similar mechanism has been reported in ERBB2-positive breast cancer. In this setting, BETi re-sensitize breast cancer cells become resistant to ERBB2 inhibitor lapatinib, by suppressing the expression of alternative RTKs, including ERBB3, IGF1R, DDR1, MET, and FGFR [177].

HER2 receptor aberrations, including mutations and amplifications, have been shown as a mechanism of resistance to EGFR inhibitors in NSCLC that can be targeted by third generation RTKi osimertinib. Treatment with BETi enhances osimertinib anti-tumor activity against HER-mutated NSCLC [178]. In the NSCLC setting, BETi can also enhance the activity of DDR2 inhibitor dasatinib in a model of DDR2-induced tumorigenesis [179].

BETi can synergize with BRAF inhibitors (BRAFi) in melanoma and colorectal cancer models and can re-sensitize BRAF-mutant cancer cells become resistant to BRAFi [180,181,182]. Ma et al showed that a combination of BETi and MEKi reduced c-MYC protein levels and induced significant tumor regression in either KRAS-mutated or BRAF-mutated colorectal cancer in vivo models [183]. A similar synergistic effect has been shown also in ovarian cancer model [184]. Wyce et al have shown that the presence of KRAS mutations is a biomarker of resistance to BETi in a panel of cancer cell lines and that combined treatment with MEKi resulted in a synergistic effect of the two drugs [185]. In line with these results, Echevarria-Vargas et al demonstrated that BETi and MEKi synergistically inhibit the growth of N-RAS mutant melanoma by inducing cell cycle perturbations and apoptosis through downregulation of TCF19 transcription factor [186].

Treatment with BETi CPI203 has been shown to increase the antitumoral activity of rapamycin through c-MYC downregulation both in vitro and in vivo in a model of pancreatic neuroendocrine tumor [187]. A similar effect was observed with JQ1 and rapamycin in a model of osteosarcoma, but the effect of JQ1 was mediated by the downregulation of RUNX2 [188]. In hematological malignancies, OTX-015 has been shown to target, in addition to c-MYC, also the NFKB/TLR/JAK/STAT signaling and to synergize with everolimus in B-cells lymphoma in vitro and in vivo [189,190]. An addictive effect of OTX-015 with everolimus has been reported also in cell models of triple negative breast cancer (TNBC) [191].

Finally, BETi have shown also a cooperative activity with multi-kinase inhibitor ponatinib in a panel of solid tumors cell lines (colon, breast, and ovary) [192].

A phase I/II clinical trial for the treatment of SCLC and other RAS-mutated solid tumors with I-BET762 in combination with MEKi trametinib is currently ongoing.

### 5.9. Combination of BETi with Hormone Therapy

BRD4 has been shown to directly interact with AR to promote AR-mediated transcriptional program. Treatment with JQ1 or I-BET762 is able to impair AR signaling and demonstrated pre-clinical efficacy in castration-resistant prostate carcinoma [193].

BETi can also downregulate ERα expression, disrupting ERα signaling and impairing the growth of tamoxifen-resistant breast cancer [194,195].

Several clinical trials are currently ongoing, testing the safety and the efficacy of combining different BETi with hormone therapy in both prostate and breast cancer settings.

### 5.10. Combination of BETi with CDK Inhibitors

Cyclin-dependent kinases (CDK) are a family of serine/threonine kinases regulating cell cycle progression and other cellular processes, including transcription and mRNA processing. Several inhibitors of these proteins with different specificity have been developed as anti-cancer agents, including palbociclib (CDK4/6 inhibitor), flavopiridol, and dinaciclib (CDK9 inhibitors), milciclib (CDK2 inhibitor), SNS032 (CDK2/7/9 inhibitor), and THZ1 (CDK7 inhibitor) [196].

CDK9 inhibitors have been shown to synergize with BETi in rhabdoid tumors and osteosarcoma through a c-MYC-dependent and a c-MYC-independent mechanism respectively [197,198]. CDK9-mediated phosphorylation can reactivate AR signaling in castration-resistant prostate cancer. In this setting, a combination of BETi and CDK9 inhibitors has shown efficacy [199].

In brain tumors, CDK1 and CDK2 promote oncogenesis by phosphorylating and stabilizing MYCN. In line with these findings, in a model of medulloblastoma, Bolin et al. showed that CDK2 inhibitor milciclib strongly synergized with BETi in downregulating c-MYC, leading to cell cycle arrest and apoptosis and to prolonged survival of mice orthotopically injected into the cerebellum with medulloblastoma cells [200].

In MYCN-dependent neuroblastoma, Durbin et al. demonstrated that a small number of transcription factors are required to maintain MYCN-amplified neuroblastoma and that this transcription circuitry can be inhibited by a combination of BETi and THZ1, being the effect of these drugs synergistic both in vitro and in vivo [201].

In a recent paper, Tomska et al systematically tested combinations of 32 drugs in a panel of blood cancer cell lines. They found that OTX-015 has synergistic effects with 41% of the tested compounds and the strongest interaction was obtained by combining OTX-015 with SNS032 in Burkitt Lymphoma cells [202].

### 5.11. Combination of BETi with BCL-2 Inhibitors

BCL-2 family proteins regulate intrinsic apoptosis, being the founder BCL-2 a potent anti-apoptotic and pro-survival factor. Abrogation of activity of anti-apoptotic BCL-2 family members through BH3 mimetics has been proposed as anti-cancer therapeutic strategy in both hematologic and solid tumors. Notably, in 2016 BH3 mimetic venetoclax has been approved by FDA for chronic lymphocytic leukemia and many clinical trials are undergoing as monotherapy or in combination with other drugs for different hematological and solid neoplasias [203].

A drug screening conducted on T-cell acute lymphoblastic leukemia primary samples revealed a synergistic activity between venetoclax and JQ1. This activity has been confirmed in xenograft models and has been ascribed to the induction of the pro-apoptotic factor BCL2L11 and concomitant reduction of BCL-2 upon JQ1 treatment [204]. The modulatory effect on the intrinsic apoptotic pathway has been described also for BETi ABBV-075. This drug has been shown to synergize with venetoclax in pre-clinical models of MM and AML [205]. In the setting of solid tumors, a similar synergistic activity of BETi and BH3 mimetics has been reported also in malignant glioma and SCLC models [206,207].

Phase I clinical trials are ongoing to test the combination of TEN-010 and ABBV-075 with venetoclax in selected hematological and solid tumors.

### 5.12. Combination of BETi with Immune Checkpoint Inhibitors

BETi have been reported as immune-modulatory agents, having a general anti-inflammatory effect and a specific repressive effect on interferon-stimulated genes. Two recent papers showed that BETi can increase the efficacy of both anti-PD1 and anti-CTLA4 monoclonal antibodies. In particular, in a KRAS-mutated NSCLC model, it has been shown that BETi remodel the cancer immune microenvironment, by reducing tumor-infiltrating Treg and inducing T-helper type 1 lymphocytes [208,209].

Two clinical trials are currently ongoing to test the combination of TEN-010 and BMS-986158 with checkpoint inhibitors atezolizumab and nivolumab, respectively, for the treatment of solid malignancies.

## 6. Conclusions

The knowledge of epigenetic mechanisms occurring during cancer development has expanded the number of possible targets and pharmacological strategies. In the last years, HDACi have proven efficacy in hematological malignancies and entered clinical practice, while efficacy of BETi is still under evaluation. However, the great potential of these classes of compounds is their capacity to interfere with basically any key pathway in cancer, through a number of different and context-dependent mechanisms. This property can be exploited by rationally combining these drugs with each other or with other classes of anti-cancer drugs, with the aim to increase their efficacy and overcome innate or secondary resistance.

## Figures and Tables

**Table 1:** Summary of published clinical trials evaluating HDAC inhibitors (HDACi) in combination with other anti-cancer compounds.

HDAC Inhibitor	Class of Combination Drug	Intervention	Condition	Phase	NCT Number	Reference
Vorinostat	Chemotherapy	Vorinostat, paclitaxel, carboplatin	NSCLC	II	NCT00481078	[106]
	Chemotherapy	Vorinostat, paclitaxel, carboplatin	Adult Solid Tumor	I	NCT00287937	[105]
	Chemotherapy	Vorinostat, doxorubicin	Adult Solid Tumor	I	NCT00331955	[108]
	Radiotherapy	Vorinostat, radiotherapy	Pelvic Cancer	I	NCT00455351	[109]
	Chemotherapy, radiotherapy	Vorinostat, capecitabine, radiotherapy	Pancreatic Cancer, Periampullary Adenocarcinoma	I	NCT00983268	[112]
	Proteosome inhibitor	Vorinostat, marizomib	NSCLC, Pancreatic Cancer, Melanoma, Lymphoma, MM	I	NCT00667082	[113]
	Proteosome inhibitor	Vorinostat, bortezomib	MM	I	NCT00858234	[114]
	Proteosome inhibitor	Vorinostat, bortezomib	Glioblastoma, Gliosarcoma, Recurrent Adult Brain Tumor	II	NCT00641706	[115]
	RTKi	Vorinostat, ridaforolimus	Lymphoma, Unspecified Adult Solid Tumor	I	NCT01169532	[116]
	RTKi	Vorinostat, sirolimus, everolimus, temsirolimus	Advanced Cancer	I	NCT01087554	[117]
	Hormone therapy	Vorinostat, tamoxifen	Breast Cancer	II	NCT00365599	[118]
Valproic Acid	Chemotherapy	Valproic acid, epirubicin,5-fluorouracil, cyclophosphamide	Advanced neoplasms	I	NCT00246103	[107]
	Chemotherapy, radiotherapy	Valproic Acid, temozolomide, radiation therapy, adjuvant therapy	High Grade Gliomas, Brain Tumors	II	NCT00302159	[101]
	Chemotherapy, radiotherapy	Valproic acid, capecitabine, radiotherapy	Colorectal Cancer	I/II	NCT01898104	[111]
Panobinostat	Radiotherapy	Panobinostat, radiotherapy	Recurrent Glioma, High-grade Meningioma, Brain Metastasis	I	NCT01324635	[110]
	RTKi	Panobinostat, erlotinib	Lung Cancer, Head and Neck Cancer	I	NCT00738751	[119]
	Hormone therapy	Panobinostat, bicalutamide	Prostate Cancer	I/II	NCT00878436	[120]
Romidepsin	RTKi	Erlotinib, romidepsin	Lung Cancer, Metastatic Cancer	I/II	NCT01302808	[121]
Etinostat	RTKi	Etinostat, sorafenib	Advanced or Metastatic Solid Tumors, refractory or relapsed AML	I	NCT01159301	[122]
	Immunotherapy	Etinostat, aldesleukin (IL-2), radiotherapy	Clear Cell Renal Cell Carcinoma	I/II	NCT01038778	[123]
Ricolinostat	Proteosome inhibitor	Ricolinostat, bortezomib, dexamethasone	MM	I/II	NCT01323751	[124]

**Table 2:** Summary of published and ongoing clinical trials evaluating BETi as monotherapy or in combination with other anti-cancer compounds.

BET Inhibitor	Class of Combination Drug	Intervention	Condition	Phase	NCT number	Reference
I-BET762 (GSK525762 or molibresib)	Monotherapy	GSK525762	Relapsed refractory hematological malignancies	I/II	NCT01943851	
	Monotherapy	GSK525762	NUT Midline Carcinoma and other Solid Cancers	I/II	NCT01587703	
	Monotherapy	Molibresib	Compassionate use in NUT Midline Carcinoma		NCT03702036	
	Hormone therapy	GSK525762; fulvestrant	HR+/HER2− advanced or metastatic breast cancer	I/II	NCT02964507	
	Hormone therapy	GSK525762; abiraterone; enzalutamide; prednisone	Castrate-resistant prostate cancer	I	NCT03150056	
	MEK inhibitors	GSK525762; trametinib	SCLC and RAS-mutated solid tumors	I/II	NCT03266159	
OTX-015 (MK-8628)	Monotherapy	OTX-015	Hematological malignancies	I	NCT01713582	[67,68]
	Monotherapy	MK-8628	Glioblastoma multiforme	II	NCT02296476	
	Monotherapy	MK-8628	Advanced solid tumors	I	NCT02698176	
	Monotherapy	MK-8628	Advanced solid tumors	I	NCT02259114	
	Monotherapy	MK-8628	Hematological malignancies	I	NCT02698189	
	Chemotherapy	OTX-015; azacitidine	AML	I/II	NCT02303782	
TEN-010 (RO6870810)	Monotherapy	RO6870810	Advanced solid tumors	I	NCT01987362	
	Monotherapy	RO6870810	AML and myelodysplastic syndrome (MDS)	I	NCT02308761	
	Immune checkpoint inhibitors	RO6870810; atezolizumab	TNBC and/or ovarian cancer	I	NCT03292172	
	Anti CD38	RO6870810; daratumumab	Advanced MM	I	NCT03068351	
	BCL2 inhibitor	RO6870810; venetoclax; rituximab	Diffuse large B-cell lymphoma and/or high-grade B-cell lymphoma	I	NCT03255096	
CPI-0610	Monotherapy	CPI-0610	Malignant Peripheral Nerve Sheath Tumors	II	NCT02986919	
	Monotherapy	CPI-0610	MM	I	NCT02157636	
	Monotherapy	CPI-0610	Progressive lymphoma	I	NCT01949883	
	JAK inhibitor	CPI-0610; ruxolitinib	Acute Leukemia, Myelodysplastic Syndrome, Myelodysplastic/Myeloproliferative Neoplasms, and Myelofibrosis	I/II	NCT02158858	
FT-1101	Chemotherapy	FT-1101; azacitidine	AML or non-Hodgkin Lymphoma	I	NCT02543879	
INCB054329	Monotherapy	INCB054329	Advanced Solid Tumors and Hematologic Malignancy	I/II	NCT02431260	
GSK2820151	Monotherapy	GSK2820151	Advanced or recurrent solid tumors	I	NCT02630251	
ZEN003694	Monotherapy	ZEN003694	Metastatic Castration-Resistant Prostate Cancer	I	NCT02705469	
	Hormone therapy	ZEN003694; Enzalutamide	Metastatic Castration-Resistant Prostate Cancer	I/II	NCT02711956	
BMS-986158	Immune checkpoint inhibitors	BMS-986158; Nivolumab	Selected advanced cancers	I/II	NCT02419417	
ABBV-075	BCL2 inhibitor	ABBV-075; Venetoclax	Selected hematological and solid cancers	I	NCT02391480	
GS-5829	Hormone therapy	GS-5829; Exemestane; Fulvestrant	Advanced Estrogen Receptor Positive, HER2 Negative-Breast Cancer	I/II	NCT02983604	
	Hormone therapy	GS-5829; Exemestane; Fulvestrant	Advanced solid tumors and lymphomas	I	NCT02392611	
	Hormone therapy	GS-5829; Enzatulamide	Metastatic Castrate-Resistant Prostate Cancer	I/II	NCT02607228	
PLX51107	Monotherapy	PLX51107	Advanced solid and hematologic malignancies	I/II	NCT02683395	
